# Development of Personalized Therapeutic Strategies by Targeting Actionable Vulnerabilities in Metastatic and Chemotherapy-Resistant Breast Cancer PDXs

**DOI:** 10.3390/cells8060605

**Published:** 2019-06-18

**Authors:** Simona Punzi, Marine Meliksetian, Laura Riva, Federica Marocchi, Giancarlo Pruneri, Carmen Criscitiello, Franco Orsi, Lorenzo Spaggiari, Monica Casiraghi, Paolo Della Vigna, Lucilla Luzi, Giuseppe Curigliano, Pier Giuseppe Pelicci, Luisa Lanfrancone

**Affiliations:** 1 Department of Experimental Oncology, European Institute of Oncology IRCCS, 20139 Milan, Italy; simona.punzi@ieo.it (S.P.); marine.meliksetyan@ieo.it (M.M.); federica.marocchi@ieo.it (F.M.); giancarlo.pruneri@istitutotumori.mi.it (G.P.); carmen.criscitiello@ieo.it (C.C.); franco.orsi@ieo.it (F.O.); lorenzo.spaggiari@ieo.it (L.S.); monica.casiraghi@ieo.it (M.C.); paolo.dellavigna@ieo.it (P.D.V.); lucilla.luzi@ieo.it (L.L.); giuseppe.curigliano@ieo.it (G.C.); piergiuseppe.pelicci@ieo.it (P.G.P.); 2 Center for Genomic Science of IIT@SEMM, Fondazione Istituto Italiano di Tecnologia (IIT), 20139 Milan, Italy; lr15@sanger.ac.uk; 3 Wellcome Sanger Institute, Wellcome Genome Campus, Hinxton, Cambridge CB10 1SA, UK; 4 Department of Oncology and Hemato-Oncology, University of Milano, 20140 Milan, Italy

**Keywords:** breast cancer, PDX, oncogenic alterations, personalized therapies

## Abstract

Human breast cancer is characterized by a high degree of inter-patients heterogeneity in terms of histology, genomic alterations, gene expression patterns, and metastatic behavior, which deeply influences individual prognosis and treatment response. The main cause of mortality in breast cancer is the therapy-resistant metastatic disease, which sets the priority for novel treatment strategies for these patients. In the present study, we demonstrate that Patient Derived Xenografts (PDXs) that were obtained from metastatic and therapy-resistant breast cancer samples recapitulate the wide spectrum of the disease in terms of histologic subtypes and mutational profiles, as evaluated by whole exome sequencing. We have integrated genomic and transcriptomic data to identify oncogenic and actionable pathways in each PDX. By taking advantage of primary short-term in vitro cultures from PDX tumors, we showed their resistance to standard chemotherapy (Paclitaxel), as seen in the patients. Moreover, we selected targeting drugs and analyzed PDX sensitivity to single agents or to combination of targeted and standard therapy on the basis of PDX-specific genomic or transcriptomic alterations. Our data demonstrate that PDXs represent a suitable model to test new targeting drugs or drug combinations and to prioritize personalized therapeutic regimens for pre-clinal and clinical tests.

## 1. Introduction

Breast cancer is the second most common cancer overall and it is the most frequent cancer in women worldwide [1]. Human breast cancer is well recognized as a heterogeneous disease characterized by distinct histology, genomic alterations, gene expression patterns, and metastatic behavior, which all deeply influence prognosis and treatment responses [2,3,4].

Gene expression profiling has been largely used for breast cancer prognosis, and it has allowed classification into five intrinsic subtypes with distinct clinical outcomes, which map to immunochemistry-defined categorization: (1) luminal A (estrogen receptor alpha, ER+, and progesterone receptor positive, PgR+; ERBB2 negative, HER2− and negative for the proliferation antigen Ki67; 24% of cases); (2) luminal B (ER+PgR+, ERBB2(HER2)+ or −, Ki67+; 53%); (3) HER2-type (HER2+, ER−, PgR−; 11%); (4) basal-like (ER− PgR− HER− triple-negative; 11%); and, (5) Normal-like (ER+ PgR+ HER2−, Ki67-; 8%) [5,6,7].

This classification is also informative for treatment choices in breast cancer patients. Targeting the ER by selective ER modulators (SERM) or reducing estradiol with aromatase inhibitors (AI) is the main treatment of ER+ breast cancer, but endocrine therapy resistance eventually occurs, which leads to disease progression. Intensive research has been undertaken to decipher the mechanism of SERM and AI resistance, resulting in the identification of complex pathways, including the modulation of ER signaling through ESR1 mutation, up regulation of growth factor receptors (HER2, EGFR, FGFR, IGFIR), alterations of the PI3K-PTEN/AKT/mTOR pathway, and NFkB signaling [8,9]. Patients with ERBB2-positive tumors receive the ERBB2-targeted antibody or small-molecule inhibitor therapy combined with chemotherapy [10]. Instead, there are no targeted therapies for the Triple Negative (TN) subtype, and current regimens involve taxane- or anthracycline-based therapies in either neoadjuvant or adjuvant settings. Additionally, in this case, the patients frequently progresses to metastatic disease, but the molecular mechanisms underlying the development of drug resistance remain largely unclear [11]. Also, drug combination is a widely used approach to decrease side-effects and enhance the therapeutic efficacy at the same time [12].

The high failure rate in the treatment of breast cancer parallels the high inter-patient genetic and phenotypic heterogeneity of this tumor, the poor mechanistic understanding of specific vulnerabilities within individual patients and the lack of preclinical models with predictive value. Thus, there is an urgent need for experimental models that replicate the heterogeneity of breast cancer at individual levels in a preclinical setting. Patient-derived xenograft (PDX) models represent the full spectrum of such heterogeneity in terms of inter-tumor diversity, since they resemble the genetic and transcriptomic profiles of the patients, and even of intra-tumor variety, where the clonal and cellular diversity of breast cancer cell populations are shown [13]. As a result, the PDX models exhibit a similar response to anti-cancer drugs and they represent a powerful tool for translational research. In fact, multiple therapies can be tested in PDXs that are generated from naïve- and post- treatment patients, faithfully representing susceptibility and resistance to therapeutic regimens [14]. For these reasons, diverse academic institutions have developed PDX platforms to investigate mechanisms of acquired resistance, to identify biomarkers of breast cancer biology, and to design new drug treatments [15].

In the present study, we took advantage of our collection of breast cancer PDXs, which were created from metastatic breast cancer samples, to uncover tumor frailties by coupling transcriptome and genomic data. The integration of genetic and transcriptomic information at the level of single tumors proved to be a valid approach in the identification of specific actionable vulnerabilities, which could be tested as predictive markers for targeted treatments, alone or in combination.

## 2. Materials and Methods

### 2.1. Sample Collection and Ethic Statements

The European Institute of Oncology provided three normal and 23 breast cancer tissues and blood of breast cancer patients (Milan). Only patients presenting with metastatic disease and with previous chemotherapy or hormonal therapy were included in the study. Breast tumor/normal tissues were collected from 2012 to 2015. Investigations have been conducted in accordance with the ethical standards and according to National and International guidelines. In vivo studies were performed after approval from our fully authorized animal facility, notification of the experiments to the Ministry of Health (as required by the Italian Law) (IACUCs Nº 757/2015), and in accordance to European Union (EU) directive 2010/63. Human tissue biopsies were collected from patients whose informed consent was obtained in writing according to the policies of the Ethics Committee of the European Institute of Oncology and regulations of Italian Ministry of Health. The studies were conducted in full compliance with the Declaration of Helsinki.

### 2.2. PDX Tissue Bank Generation

Patients that were enrolled in the study were selected on the basis of highly aggressive metastatic disease diagnoses (Luminal B, Triple Negative and HER2+ subtypes) and resistance to different lines of therapy. Patient tumor biopsies from liver (*n* = 12), lung (*n* = 8), and axillary lymph node (*n* = 3) were transplanted in the fourth mammary gland of female NSG mice (*n* = 3), together with Matrigel (Corning #356231). No enzymatic or mechanical tumor dissociation was performed at the first passage in mice. The animals were monitored for engraftment by routine palpation and the tumors were harvested when they reached a volume of 0.8 cm^3^. 12 out of 23 tumors efficiently engrafted in mice at their first passage. Subsequently, the tumors were digested by enzymatic and mechanical digestion (Miltenyi Biotec) and 5 × 10^5^–1 × 10^6^ cells were resuspended in Matrigel/PBS (1:1) and orthotopically transplanted as above in NSG mice (*n* = 10–15). The same protocol was applied for further passages in mice, up to three (MBC2, MBC3, MBC4, MBC5, MBC7, MBC10, MBC11, MBC18, MBC21, MBC22 and MBC26) or four (MBC1) serial transplantations. PDX tumors were also frozen as fragments or cell suspension for additional experimental purposes or re-transplantation.

### 2.3. Animals

NOD.Cg-Prkdc^scid^ Il2rg^tm1Wjl^/SzJ (NSG) mice were purchased from Charles River. Female mice 6–12 weeks old (15–20 g weight) were used for experimental procedures.

### 2.4. PDX Culture

For in vitro assays, primary two-dimensional (2D) culture of PDX cells (PDXC) were generated by plating single cell suspension of tumors grown in the animal. The cells were obtained by enzymatic and mechanical digestion, as described above. PDXCs were maintained in culture for a short period of time (3 days) in DMEM/F12 (1:1, Lonza/Gibco) supplemented with 10% Fetal Bovine Serum (FBS) (HyClone, GE Healthcare Life Science, Pittsburgh, PA, USA), 10mM HEPES (Sigma Aldrich-Merck KGaA, Darmstadt, Germany), 5 μg/mL insulin (Roche, Basel, Switzerland), 0.5 μg/mL hydrocortisone (Sigma Aldrich-Merck KGaA, Darmstadt, Germany), 10 ng/mL epidermal growth factor (EGF, Tebu-Bio, Le Perray En Yvelines, France), and 50 ng/mL Cholera Toxin (Sigma Aldrich-Merck KGaA, Darmstadt, Germany). PDXC were maintained in aforementioned culture conditions to perform drug testing (see Section 2.7).

### 2.5. Exome-Sequencing

Genomic DNA (gDNA) of patients’ samples was extracted from frozen (MBC1, MBC3, and MBC5) or formalin-fixed, paraffin-embedded (FFPE) (MBC2) tissues, containing at least 50% breast cancer cells tissues, as confirmed by the pathologist. gDNA was also obtained from the blood of matched patients’ tumor tissues (normal counterpart) and xenograft at different passages (all from frozen tissues). gDNA was prepared while using the Quiagen DNeasy Blood & Tissue Kit, fragmented (Quiagen, Cluj-Napoca, Romania) and used for Illumina Truseq library construction. Exome-capture was performed using the SureSelectXT Human All Exon Kit (version 4), according to the manufacturer’s instructions (Agilent Technologies, Santa Clara, CA, USA). Whole-exome sequencing was performed with the Illumina Hiseq 2000 (Illumina Inc., San Diego, CA, USA) platform with 101 bp paired-end reads. Sequencing alignment and subsequent bioinformatic analysis were performed, as previously described [16].

### 2.6. RNA-Sequencing

Total RNA was extracted from three normal breast tissues and MBC2, MBC7, MBC3, and MBC26 PDXs cells by using Zymo Research RNA extraction kit (Freiburg im Breisgau, Germany), at the latest passage (PDX3) analyzed for the genomic profile and used to perform drug testing. mRNA purification and NGS libraries were obtained following Illumina instruction (TruSeq RNA Sample Preparation). 50 bp paired-end RNA-seq reads were aligned to the genome (hg19, GRCh38) while using TopHat2 2.0.9 [17]. Read counts of each gene were quantified using HTseq [18] and differential analysis was performed while using DESeq or edgeR Bioconductor packages [19,20]. Gene set enrichment analysis was performed using GSEA (Gene Set Enrichment Analysis-Broad Institute, Inc. Cambridge, MA, USA) software v2.2.0 (www.broadinstitute.org/gsea/index.jsp) with GO biological process MSigDB gene sets using default parameters. Gene sets enriched at False Discovery Rate (FDR) < 0.25 were considered to be significant.

### 2.7. Drug Test

Short-term in vitro growth inhibition by drugs in PDX cells was assessed by Cell Titer Glo (Promega). Briefly, PDXC (obtained from third passage in mice) were thawed and used as primary 2D in vitro culture. The cells were plated in 96 wells (5000 cells per well) and treated for three days by a single and continuous exposure to vehicle or increasing concentrations of the following drugs: standard therapies—Paclitaxel (0.5–2.5 μM), or 4-Hydroxytamoxifen (0.1–12 μM) -targeted therapies -Idasanutlin (5–15 μM-Selleckchem) or Everolimus (5–25 μM-MCE)—alone or in combination. The inhibitory concentrations (IC_30_ and IC_50_) and percent viability inhibition by fixed doses of the aforementioned drugs were calculated while using GraphPad Prism software (GraphPad Software Inc. San Diego, CA, USA).

### 2.8. Western Blotting

Western blotting was performed after 24 h treatments of PDXC. The cells were lysed in RIPA buffer and processed, as previously described [21]. Membranes were probed with the following antibodies: p21 (Santa Cruz Biotechnology, Dallas, TX, Texas), phospho-S6K (p-S6K) (Elabscience, WuHan, China), phospho-S6 (p-S6) (Cell Signaling Technology, London, UK), γH2Ax (Biolegend, San Diego, CA, USA), PCNA (Dako, Santa Clara, CA, United States) and cleaved PARP (Cell Signaling Technology, London, UK). Histone H3 (Abcam, Cambridge, UK) or Vinculin (Sigma Aldrich-Merck KGaA, Darmstadt, Germany) were used as normalizers. The images were cropped at specific protein band of interest to improve the clarity of data presentation.

### 2.9. Statistical Analysis

The data are represented as mean ± SD of biological triplicates. Comparisons between drug efficacy among groups were assessed by using one-way ANOVA followed by Dunnett Post test. *p* ≤ 0.05 and lower were considered significant.

### 2.10. Data Access

Data sets are available in the Gene Expression Omnibus (GEO) database under accession number GSE129563.

## 3. Results

### 3.1. Breast Cancer PDXs Recapitulate the Biology of the Tumor of Origin

**Figure 1:**
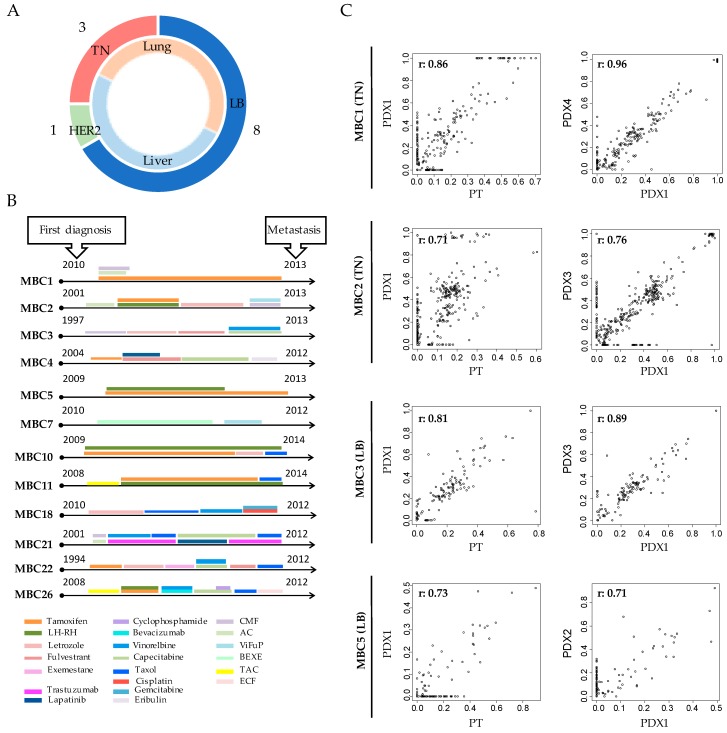
Patient-derived xenograft (PDXs) resemble histologic and mutational features of the corresponding patient. Patient Derived Xenografts (PDXs) were obtained by the transplantation of patients’ (PTs) tumor in the 4th mammary gland of immunocompromised mice. (**A**) Doughnut represents the number of PDXs divided *per* subtype (LB: Luminal B; TN: Triple Negative; HER2 positive: HER2) in the external circle and site of PT’s metastasis in the inner one. (**B**) Time lines indicate the year of the first diagnosis and of the metastasis transplantation in mice to create the corresponding PDX. Colored bars indicate the therapeutic regimens administered to each PT. CMF: Cyclophosphamide-Methotrexate-Fluorouracil; AC: Adriamycin-Cyclophosphamide; ViFuP: Vinorelbine-Fluorouracil-Cisplatin; BEXE: Bevacizumab-Capecitabine-Cyclophosphamide-Erlotinib; TAC: Docetaxel-Adriamycin-Cyclophosphamide; ECF: Epirubicin-Cisplatin-Fluorouracil. (**C**) Whole Exome sequencing was performed in four PTs and PDXs. Scatter plots of mutations identified in each PT and corresponding PDX at different passages are reported. In the left panels the x-axis indicates Variant Allele Frequency (VAF) of the mutations present in the PT and the y-axis indicates those present in the paired PDX at first passage (PDX1). In the right panel the x-axis indicates VAF of PDX1 and the y-axis the VAF of the latest passage obtained for that PDX. Pearson correlations (r) are reported.

We established a collection of PDXs derived from a specific setting of high incidence relapsed patients in order to create clinically relevant mouse models of human breast cancer. In details, biopsies from liver (*n* = 12), lung (*n* = 8) and axillary lymph node (*n* = 3) metastasis were obtained from patients with different breast cancer subtypes, according to the intrinsic classification in Luminal B; Triple Negative and HER2+ (Table S1). As first passage, the tumors were transplanted as fragments in the fourth mammary gland of NSG mice. 12 out of 23 transplanted tumors (52%) efficiently engrafted in mice: 8/15 Luminal B (LB), 1/3 HER2+, and 3/5 TN (Figure 1A and Table S1). Patients with HER2+ BC were underrepresented in our collection, consistently with their lower patient frequency and engraftment rate [15]. Successful tumor growth did not correlate with their hormonal status (ER or PgR), or with metastasis site (liver or lung). LB showed high engraftment capability, as compared to published reports [22]. After the first passage, tumors were serially re-transplanted as cell suspension to obtain the propagation and expansion of each sample in subsequent passages.

Tumors from patients and corresponding PDXs were characterized while using the classical immunohistochemistry (IHC) markers ER, PgR, HER2, and Ki67. All PDXs retained the same features of the original tumors (Table S1 and Figure S1A,B for representative staining of TN–MBC7- and LB–MBC22 samples). Cells that were positive for the Human Leukocyte Antigen (HLA) were also Pan-cytokeratin -positive, thus demonstrating that the growing tumors were of human origin. Expectedly, both the ductal layer composed of myoepithelial cells and the stroma were HLA-negative and α-SMA positive (Figure S1C), since human stroma is lost during engraftment and is replaced by the murine one [23]. Moreover, all of the PDXs were derived from patients who developed resistance to endocrine and/or chemotherapies (Figure 1B).

We performed whole exome sequencing (WES) of four different PDXs (2 TN: MBC1 and MBC2 and 2 LB: MBC3 and MBC5), the corresponding metastasis of origin, and the blood of the patients as normal counterpart to verify that our PDXs also retained the genomic features of the original tumors (Table S2). We applied a robust computational pipeline to discriminate human and mouse sequencing-reads due to variable amounts of murine cells composing the stroma, which allowed for restricting analyses to human component of our tumor samples [16]. At the genomic level, the PDXs showed a similar mutational profile in terms of type of mutations (single nucleotide variants; SNVs) and their variant allele frequency (VAF ≥ 0.05), both with respect to the corresponding primary tumor-sample (Figure 1C left panels; Pearson correlations: r = 0.71–0.86) and after re-transplantation (Figure 1C, right panels; r = 0.71–0.96). Snapshots from two different PDXs are reported in Supplementary Figure S1D, showing the persistence of patient somatic mutations (a missense mutation in MBC1 and a nonsense mutation in MBC2) or a germline variant in the PDX MBC1 (highlighted in blue). About 3–7% of alterations of unknown significance were unique to the PT (Table S2). A higher number (about 4–20%) of mutations, though rarely functionally significant, were present in PDX1, but not in the PT, which suggested a minimum drift during engraftment, as previously observed [24]. Notably, this was the case for the missense mutation of the tumor suppressor ARID1A in MBC1 and MBC2 PDXs or for GATA3 insertion in MBC3 (Table S2). The appearance of new mutations in PDX1 might be the consequence of some degree of clonal selection during in vivo growth, since mutations of ARID1A are associated with increased cellular proliferation and have been reported in a variety of human cancers [25]. Finally, Pearson correlations were extremely high among PDXs at different passages (Supplementary Figure S1E), supporting high genome stability upon serial transplantation in mice.

Overall, these results suggest that breast cancer PDXs faithfully represent the wide spectrum of phenotypic and genomic abnormalities of the disease and remain sufficiently stable when re-transplanted.

### 3.2. Each Metastatic PDX Represents a Unique Genomic Landscape Pattern

**Figure 2:**
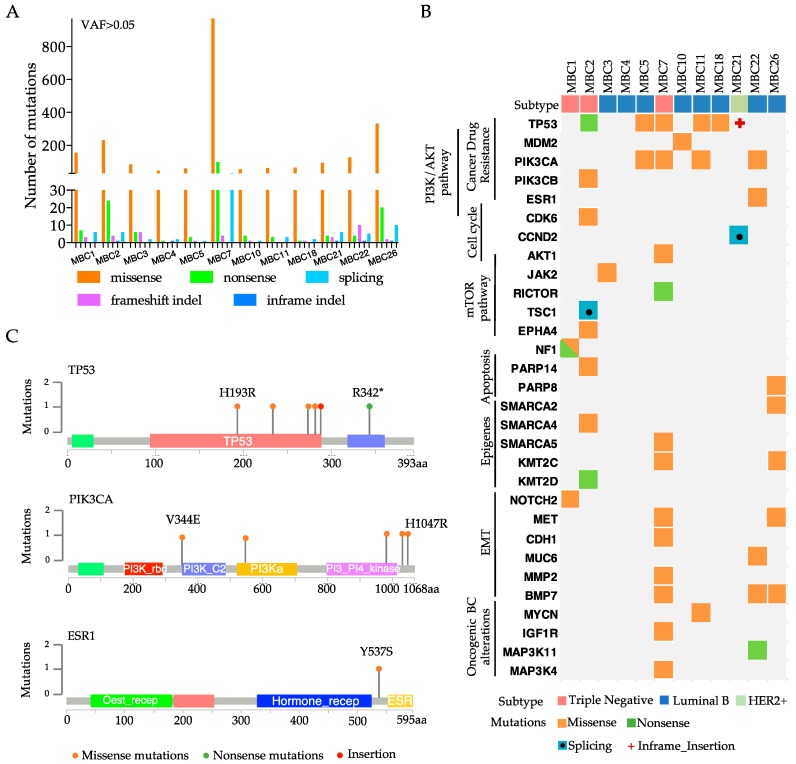
Genomic landscape of 12 PDXs embodies known driver alterations of breast cancer. (**A**) Number of total somatic mutations (with Variant Allele Frequency VAF > 0.05) obtained by Exome-sequencing analysis of each PDX. Colored bars represent Single Nucleotide Variants (Missense or Nonsense mutations), Insertion/deletion (InDel) or splicing. (**B**) Somatic alterations including most of the major breast cancer driver events in each PDX are represented in the heatmap. On the top, molecular subtypes of each PDX are reported. (**C**) Lollipop diagrams of annotated genetic variations in TP53, PIK3CA, or ESR1 gene in the PDXs.

Since our four PDXs showed a remarkable degree of genomic fidelity with their tumors of origin, we sequenced the whole exome of other eight PDXs. The most frequent alterations that were found in the 12 PDXs were SNVs (mainly missense mutations), followed by Splicing mutations and Insertion/Deletion (InDels) (Figure 2A), which involved known driver genes and functional pathways previously described in breast cancer [26] (Figure 2B).

Expectedly, the mutations of TP53 or genes of the PI3K/AKT/mTOR and epithelial-to-mesenchymal transition (EMT) pathways were found at relatively high frequencies. Additionally, we identified the alterations of epigenetic targets, such as SWI/SNF related genes (SMARCA2, SMARCA4, SMARCA5) or histone modifiers (KMT2C or KMT2D), breast cancer-associated oncogenes (MYCN or MAPKs), or genes that are involved in drug-resistance (ESR1 and PIK3CA/B). The highest mutation rate was found in the TP53 gene (about 50%) (Figure 2B), which was slightly higher than that reported in breast cancer metastatic samples (in cBioPortal—http://www.cbioportal.org) (Figure S2A), probably due to the selective pressure of treatments administered to the patients. Mutation rate in the PIK3CA gene (about 30%; Figure 2B), instead, was in line with its expected frequency in breast cancer (cBioPortal) (Figure S2A). One LB PDX (MBC22) showed somatic mutation in the ESR1 gene (Figure 2B), which is frequently found to be mutated in ER+ metastatic breast cancer and has been correlated with resistance to fulvestrant treatment [27]. Six of our 12 PDXs showed alterations in several EMT genes (Figure 2B), which are known to be implicated in tumor metastasis [28]. When considering the most frequent and highly actionable mutations (TP53, PIK3CA and ESR1), each PDXs showed a different type of alteration in terms of protein change, suggesting that each PT/PDX has a unique molecular profile, and predicted therapeutic response (Figure 2C and Figure S2B).

IHC staining for TP53 and PIK3CA was performed in samples carrying wild type or mutated alleles to analyze the effects of the recorded mutations on protein expression. Wild-type TP53 PDXs (MBC3 and MBC26) showed modest protein expression. PDX samples with mutant TP53 displayed different patterns, with strong homogeneous (i.e., MBC2) or heterogeneous (i.e., MBC7) staining (Figure S2C–upper panel). IHC for PIK3CA showed heterogeneity among the samples and only partially correlated with the mutation per se, since PIK3CA was considerably expressed either in mutant (MBC22 and MBC7) and WT tumors (MBC3 and MBC26) (Figure S2C–lower panel). This may be due to alternative mechanisms of PIK3CA activation in samples with wild-type alleles [29].

### 3.3. Transcriptional Analysis of PDX Cells Parallels Mutational Profile

Mutations of cancer drivers induce global transcriptional changes [30], which have been shown to correlate with prognosis and treatment response [31]. Thus, we analyzed the transcriptome of four breast cancer PDXs, when comparing it to the transcriptome of normal tissue extracted from three mammary glands. We selected two TN and two LB PDXs harboring somatic mutations of TP53 p.H193R, PIK3CA p.1043I, and AKT1 p.Q85H (MBC7); TP53 p.342* and TSC1 (MBC2); JAK2 p.I702V (MBC3) and MET p.H60Y and p.D543E (MBC26).

**Figure 3:**
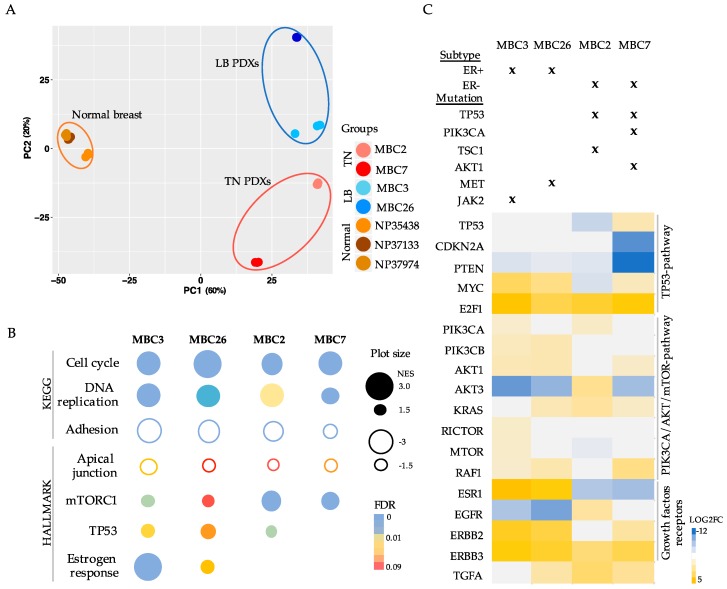
Transcriptomic/genomic profiles of breast cancer PDXs revealed alternative actionabilities. (**A**) Principal component analysis (PCA) score plot of gene expression data from RNA-seq of three normal breast tissues, two LB, and two TN PDXs (*n* = 3 replicates) with the two first principal components (PC1 and PC2) plotted on the x-axis and y-axis, respectively. (**B**) Representative Gene Set Enrichment Analysis (GSEA) based on KEGG and HALLMARK databases of each PDX in comparison with normal breast. Plot size indicates Normalized Enrichment Score (NES) (fill plots up-regulation; empty plots down-modulation); colors indicate False Discovery Rate (FDR) values. (**C**) Complementary genomic/transcriptomic data of each PDX was considered. Positivity (+) or negativity (−) to estrogen receptors (ER) is reported. Mutations of genes belonging to TP53 or PI3K/AKT/mTOR pathways are represented with an “x” in each PDX. Heatmap represents LOG2 fold change (FC) values of genes of the aforementioned pathways.

Principal Component Analysis (PCA) clearly segregated the normal from cancer samples (Figure 3A). LB and TN PDXs displayed distinct expression patterns, supporting their diverse biological origin (Figure 3A). Indeed, hierarchical clustering of the 1869 most variable genes confirmed the segregation of Normal breast, LB, and TN breast cancer PDXs (Figure S3A). As expected, the tumors showed higher heterogeneity than normal tissues, paralleling that observed in the genomic profiles.

When considering the high degree of diversity among PDXs, the transcriptome of each subject was separately analyzed when comparing it to the average of normal samples to obtain differentially expressed genes (DEGs) (Table S2). Afterwards, we conducted gene-set enrichment analysis (GSEA) of the DEGs, based on KEGG and HALLMARK databases. Among the up-regulated genes we observed enrichment of mTORC1, TP53, and estrogen-response pathways, as well as genes in “Cell cycle” and “DNA replication”, reflecting the propensity of these cells to activate unrestrained proliferative functions (Figure 3B and Figure S3B). Among the down-regulated genes, cell adhesion molecules were strongly down-modulated, as well as apical junctions, which parallels the metastatic origin of the cells (Figure 3B and Figure S3B).

We compared the transcriptome to the mutational profile in each PDX to search for tumor-specific pathways that are activated by somatic mutations. First, we confirmed that ESR1 hyper-activation was exclusively observed in ER+ tumors, while EGFR expression inversely correlated with the hormonal status (Figure 3C) [32]. ERBB2 and ERBB3 were variably expressed in the four PDXs, while the transforming growth factor alpha (TGFA) and one of its receptors (EGFR) were highly expressed in MBC2 (Figure 3C), which suggested that diverse growth factor receptors and proliferative pathways are activated, depending on the molecular subtype. As supported by GSEA analyses, all the PDXs exhibited at least one mutation in one of the up-stream regulators of the PI3K/AKT/mTOR pathway: JAK2 in MBC3, MET in MBC26, TSC1 in MBC2, and AKT and PIK3CA in MBC7, along with protein over-expression of various genes belonging to the aforementioned pathway (Figure 3C), which suggests that its specific inhibition can represent an efficient treatment opportunity.

TP53-mutated (-Mut) MBC7 showed the strongest reduction of CDKN2A and PTEN expression, which is known to inversely correlate with patient prognosis [33,34]. LB PDXs also showed the hyper-activation of MYC, whose over-expression is associated with aggressive and drug resistant phenotype [35]. Instead E2F1, whose over-expression correlates with unfavorable outcome and metastatic disease [36], was equally up-regulated in all of the PDXs (Figure 3C).

Overall, our transcriptomic data provide information on the expression levels of the genes that are downstream to the identified somatic alterations. Data integration may predict the activation of oncogenic signals and suggest alternative treatments to target tumor-specific pathways.

### 3.4. Integration of Genomic and Transcriptomic Profiles Predicts Resistance and Sensitivity to Treatments

Based on the integrated analyses of genomic and transcriptomic profiles (Figure 3C), we used the four above mentioned PDXs (MCB2, MBC7–as TN and MBC3 and MBC26–as LB), to predict drug-sensitivity to Everolimus and Tamoxifen (targeting mTOR1 and ESR1, respectively [37,38]) or drug-resistance to Idasanutlin (inactive in cells with mutated TP53 [39]). We have optimized ex vivo cultures of cells isolated from PDX tumors in order to use PDXs as a pre-clinical drug testing platform.

First, we analyzed whether our PDXC were indeed resistant to standard therapy as the corresponding patients to Paclitaxel (PTX), a drug that is used in first-line therapy of TN breast cancer and as later-line therapy in ER+ metastatic breast cancer [5]. Cells from two TN (MBC2 and MBC7) and two LB (MCB3 and MBC26) PDXs were independently treated with PTX. No PDXs responded to PTX, as compared to a positive control (MCF10DCIS BC cell line) (Figure S4A), thus resembling resistance in the corresponding patient upon treatment with PTX or other anti-mitotic agents.

**Figure 4:**
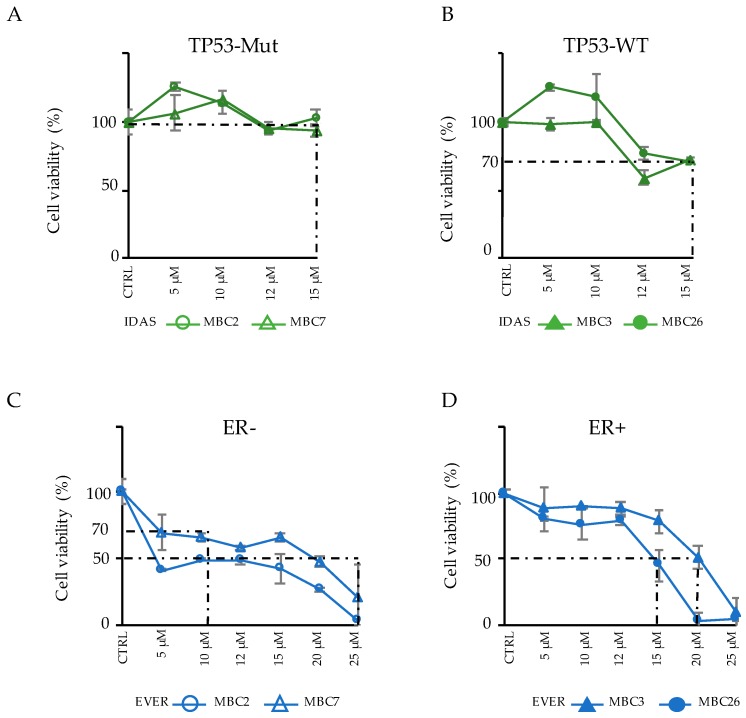
Response of PDXs to alternative drugs suggests efficient therapeutic opportunities. (**A**–**B**) TP53 mutated (Mut) (MBC2 and MBC7) (**A**) or TP53 wild-type (WT) (MBC3 and MBC26) (**B**) PDXs were treated in vitro with increasing concentration of Idasanutlin (IDAS) for three days. Response of each PDX is reported in terms of cell viability (%) with respect to control (CTRL) sample in *n* = 3 experiments (mean ± SD). (**C**–**D**) The same PDXs classified for their negativity (−) or positivity (+) to estrogen receptors (ER) were treated for three days with increasing concentrations of Everolimus (EVER). Cell viability (%) in response to the drug in the ER− (**C**) or ER+ (**D**) PDXs is reported in *n* = 3 experiments (mean ± SD).

Subsequently, we treated the same PDXC with targeting drugs, as alternative therapies. TP53-Mut and TP53-WT PDXC were treated with the TP53-MDM2 inhibitor Idasanutlin (IDAS), and cell viability was evaluated after three days (Figure 4A,B).

As expected, TP53-Mut PDXC (MBC2 and MBC7) showed resistance to IDAS (Figure 4A), while the TP53-WT cells displayed a modest sensitivity (IC_30_: 15 µM) (Figure 4B).

We then treated the same PDXC with the mTOR-inhibitor Everolimus (EVER) (Figure 4C,D). Notably MBC2 (ER-), which carries the mutation of the TSC1 gene, implicated in the mTOR pathway, showed a good response (IC_50_ = 10 µM). MBC26 (ER+) and MBC7 (ER−) cells showed a better response (IC_50_ = 12–15 µM) with respect to MBC3 (IC_50_ = 20 µM), although a comparable expression of AKT1 was detected in all three samples (Figure 3C). The ER+ tumors MBC3 and MBC26 were also tested for their response to Tamoxifen (4-Hydroxytamoxifen, 4-OHT), the elective endocrine therapy for ER+ metastatic tumors [5]. Interestingly, MBC3, which showed the highest expression of ESR1, displayed higher sensitivity to 4-OHT than MBC26 (Figure S4B, MCF-7 used as positive control).

Overall, these data confirm that PDXC reflects the drug resistance responses that were observed in the corresponding patients, and estimate the sensitivity to standard and alternative agents, thus validating our integrative analysis approach for the treatment of metastatic breast cancer patients. Paralleling mutational and expression profiles, the response to each drug is different from one PDX to the other, thus supporting the idea that combining the comprehensive genomic and transcriptomic characterization can provide an effective validation step to identify the predictive gene signatures and therapeutic response.

### 3.5. Combinatorial Drug Administration Can Prioritize Therapeutic Regimen in Patients

**Figure 5:**
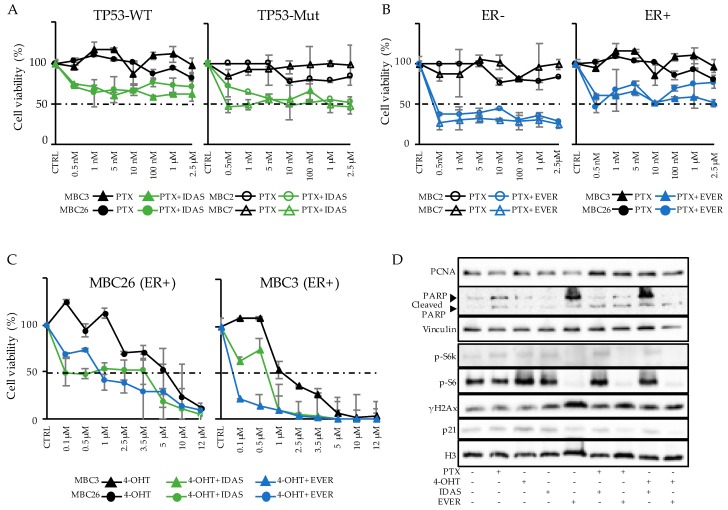
Combinatorial drug administration in PDXs is a model to uncover drug sensitivity. (**A**) Cell viability (%) in response to standard therapy with Paclitaxel (PTX) at increasing concentrations or combination with IDAS 15 µM in two TP53-Wild Type (WT; left panel) or two TP53-Mutated (Mut; right panel) PDXs is reported for *n* = 3 experiments (mean ± SD). (**B**) Cell viability (%) due to standard therapy with PTX at increasing concentrations or combination with EVER (10 µM concentration in TN tumors; 20 µM in MBC3 and 15 µM concentration in MBC26 cells) in two ER− (left panel) or two ER+ (right panel) PDXs (*n* = 3; mean ± SD). PTX response is plotted from the same experimental setting of Figure A. (**C**) 4-Hydroxytamoxifen (4-OHT) treatment at increasing concentrations in LB PDXs: MBC3 and MBC26. Combinatorial therapy is defined as combination of standard therapy with 4-OHT and alternative therapy with IDAS (15 µM) or EVER (20 µM in MBC3 and 15 µM concentration in MBC26) in *n* = 3 experiments (mean ± SD). (**D**) MBC3 was used for the evaluation of pathways modulation due to single or combinatorial drug administration. Inhibition of mTOR pathway due to EVER administration was defined by western blot analysis of phospho-S6K (p-S6K) and phospho-S6 (p-S6). Activation of DNA damage response and apoptosis were evaluated by γH2Ax and PARP cleavage, respectively. Mitotic arrest was evaluated by p21 and PCNA staining. Histone H3 and Vinculin were used as normalizers.

Drug combination has been widely considered to be a valid approach to decreasing the toxicity of specific drugs and enhancing therapeutic efficacy, exploiting their synergistic effect on tumor growth [12]. A combination of IDAS and PTX was tested in the same PDXC, as above, to uncover potential sensitization to chemotherapy following TP53 re-activation (Figure 5A). IDAS was administered at the single concentration of 15 µM, corresponding to IC_30_ in the sensitive samples (Figure 4B and Figure S5A), while PTX was added at increasing concentrations from 0.5 to 2.5 µM.

In two TP53-WT PDXC (the ER+ MBC3 and MBC26) IDAS sensitized cells exposed to scalar doses of PTX, strongly reducing proliferation with respect to non-sensitized cells (Figure 5A, left panel; MBC3: *p* < 0.001; MBC26: *p* < 0.001). Surprisingly, in two TP53-Mut PDXC (the TN MBC2 and MBC7), where IDAS per se did not affect cell viability (Figure 4A), we also observed IDAS-PTX synergy, with the combination inducing a significant reduction of cell viability quantified as more than the sum of single agents (Figure 5A, right panel; MBC2: *p* < 0.05; MBC7: *p* < 0.001).

We then tested the PTX-EVER association. The administration of EVER at a concentration of 10 µM (corresponding to IC_30_ in MBC7 and IC_50_ in MBC2; Figure 4C and Figure S5A) almost abolished cell growth in both TN ER- PDXC also at very low PTX concentrations (Figure 5B, left panel; MBC2: *p* < 0.001; MBC7: *p* < 0.001). On the contrary, EVER (IC_50_: 20 µM in MBC3 or 15 µM in MBC26) slightly reduced cell proliferation, above the effect of the corresponding IC_50_ as single agent, in two ER+ PDXs (Figure 5B, right panel–Figure S5A). Thus, EVER sensitized breast cancer cells to chemotherapy and exerted a general greater effect by reducing cell viability only in ER- PDXs.

Finally, we tested the combination of 4-OHT and IDAS or EVER in the two ER+ MBC26 and MBC3 PDXC. IDAS (15 µM concentration) and 4-OHT strongly reduced cell viability, as compared to the single 4-OHT administration (MBC26: *p* < 0.05; MBC3: P not significant—Figure 5C), even if not significant differences were detected among the standard and combination therapies in the latter (Figure S5A). EVER (20 µM in MBC3 and 15 µM in MBC26) also reduced cell viability (Figure 5C), showing additive effects due to the sum of the targeting-agent treatments with endocrine drug (MBC26: *p* < 0.05; MBC3: P value is close to statistical significance—Figure S5A).

We used one of our PDX (MBC3) to analyze the target modulation upon treatment (Figure S5B). An evaluation of drug-dependent effects can inform on the activation of alternative pathways driving therapy resistance. MBC3 cells were treated for 24h with PTX 5 nM, 4-OHT 1 µM (IC_50_), IDAS 15 µM (IC_30_), and EVER 20 µM (IC_50_) as single administration or in combination. Figure 5D reports Western blot analysis of several genes that were involved in the molecular pathways targeted by the drugs. Protein levels were normalized with respect to H3 and compared with control cells (CTRL—no treatment) (Figure S5C). Moreover, cleaved PARP and PCNA levels were used to analyze apoptosis and proliferation, respectively. PTX alone only slightly activated DNA damage (γH2Ax) and apoptosis, as expected in resistant cells. IDAS partially induced phosphorylation of H2Ax [40], which is in line with its efficacy in this PDX. EVER induced downstream inhibition of mTOR pathway, as shown by the reduction of phospho-S6K and phospho-S6, and promoted DNA damage (γH2Ax) and moderate apoptosis. IDAS exerted a synergistic effect with PTX when being administered in combination, likely activating cell cycle arrest (supported by p21 increase), DNA damage response (γH2Ax), and apoptosis (cleaved PARP). PCNA levels are increased as a result of DNA damage in the absence of cell cycling [41].

4-OHT induced p21 increase and cell death in vitro, according to the IC_50_ used. 4-OHT increased the phosphorylation levels of S6K and S6, suggesting the activation of mTOR pathway and its consequent sensitization [42]. As reported elsewhere, mTOR pathway activation may mediate ER resistance [9]. Notably, when administered in combination, 4-OHT and EVER totally abolished p-S6K and p-S6 and stimulated DNA damage and apoptosis with increased γH2Ax, PARP cleavage, and the reduction of p21 levels [43].

Together, these results demonstrate that PDXC can be tested with a large panel of targeting drugs, alone or in combination, and that agents prioritized by in vitro testing might be successfully translated in vivo to optimize the therapeutic regimens.

## 4. Discussion

In this study, we have shown the feasibility of generating breast cancer PDXs that are representative of the clinical and molecular diversity of the disease. We have demonstrated that PDXs can be extensively interrogated, both biologically and molecularly, to uncover patient-specific frailties to be therapeutically exploited. PDXs can be challenged with many candidate-targeted drugs, alone or in combination, which may be useful for the design of novel personalized treatment approaches.

The use of PDXs in pre-clinical cancer drug development has become widespread [44]. PDX models have been successfully adopted in several pre-clinical trials, since the drug response rates in PDX correlate with those that were observed in clinic. For example, it has been demonstrated that PDXs faithfully resemble clinical sensitivity to PARP-inhibitor in a pancreatic cancer model obtained from naïve or treated patients [45] or to arsenic trioxide in relapsed patients with small cell lung cancer [46]. In addition, anti-cancer drugs have been tested for precision medicine approach in ovarian cancer and leukemia PDXs [47,48]. Collectively, these efforts aim to implement personalized medicine while using in vitro and ex vivo PDX models. Our biobank involves a distinctive patients’ setting, since the PDXs were derived from metastatic samples of patients that developed resistance to standard therapeutic regimens (endocrine and/or chemotherapy). This collection of PDXs offers the unique opportunity to evaluate new drug options in the context of specific genetic alterations that have evolved under various therapeutic pressures [49], and they may thus represent the sole opportunities to prolong patients’ survival.

The comprehensive characterization of histopathological features and somatic genomic alterations confirmed that these models have a significant level of resemblance with their matched patient samples, which supports their validity for translational and pre-clinical study [23,24,31,50]. In particular, the transplantation of the patient’s tumor, either as fragments or dissociated single cells, allowed for preserving the original histologic characteristics and the vast majority of the unique molecular sub-clones that compose each individual tumor. The retention of such clonal dynamics is a key aspect in the evaluation of both tumor biological properties and efficacy of any therapeutic approach, thus suggesting that PDXs are useful for studying breast cancer biology and drug responses.

Even if the number of our cohort of PDXs is small, genomic profiling of the tumors has revealed the presence of the most frequent aberrations in breast cancer, and their unique assortment within individual samples, which suggests that each PDX is representative of the peculiar molecular portfolio of individual patients that can be used to study drug resistance and disease progression. This encompasses the idea that treating patients carrying different mutation profiles with the same drug, targeting one of the driving mutation, or combining few drugs, may be insufficient in optimizing treatment success [51].

Understanding signaling and biological effects due to somatic mutations may contribute to rationalize drug design and predict patient response [52]. Additionally, previous studies have also shown a great extent of gene expression changes in metastatic cancer cells and that, in some cases, these modifications may predict patient survival [53]. Additionally, transcriptome profiles can influence drug responses. The transcriptome analysis of our PDXs has highlighted the alterations of actionable pathways (i.e., Estrogen activation, TP53, and PI3K-AKT-mTOR) driving tumorigenic events. Beyond this, epigenetic modifiers (i.e., Methyltransferase, BET-domain, SWI/SNF, and Lysine Demethylase members) as well as known long non-coding (lnc) RNAs with a role in breast cancer progression and prognosis (i.e., MALAT1, HOTAIR1, MEG3) [54,55] have been found to be generally deregulated in both the LB and TN samples. This finding suggests that our PDX bank could be successfully used to further analyze patients’ sensitivity to many drugs, targeting different genes and pathways.

By integrating genomic and transcriptomic data, we have proposed a Patient/PDX-centric approach for the prediction of oncogenic vulnerabilities, thus prioritizing actionable alterations in breast cancer and cytotoxic drugs as a standard regimen of therapy.

Regarding the response to standard drugs, Paclitaxel resistance was reported to develop in a high percentage of breast cancer patients (30% of node-negative patients and up to 70% in node-positive BC patients), with only 23% surviving five years after diagnosis and mainly dying of metastasis spread [56]. Alike, all of the PDXs tested mirror the resistance to paclitaxel, as observed in the corresponding patients in response to the same drug or to other anti-mitotic agents. For example, MBC26 patient relapsed to Paclitaxel, MBC3 to Vinorelbine, MBC2, and MBC7 to diverse chemotherapy combinations (Vinorelbine-, Fluoruracil-, Cisplatin, or Cyclophosphamide- Methotrexate- Fluorouracil). Actually, the PDXs showed mutations in Tubulin [57] or other tumor suppressors (i.e., TP53, CDKN2A) [58], whose underlying interaction could justify the onset of anti-mitotic resistance.

The identification of alternative therapies by means of integrated genomic and transcriptomic analyses was successfully validated by in vitro drug response. As confirmed by a potent and selective MDM2-TP53 small-molecule antagonist, Idasanutlin, TP53 mutations is predictive of anti-TP53-drug resistance [59], while the treatment of cancer cells expressing functional TP53 resulted in the concurrent transcriptional activation of TP53 downstream genes, cell cycle arrest, and cell death [39].

We defined its actionability as a successful strategy to inhibit cell viability since our model highlighted the oncogenic role of PI3K-AKT-mTOR pathway alteration. Everolimus is one of the Rapamycin analogues and acts as an allosteric inhibitor of mTOR1. Genetic alterations, including mutations and/or amplifications, which activate the PI3K-AKT-mTOR pathway have been successfully targeted by Everolimus in advanced biliary tract cancer and heavily pretreated metastatic gastric cancer [60,61]. In our setting, mTOR1 inhibition triggers apoptosis in PDX carrying activating mutations of the same pathway, confirming that the mTOR1 inhibitor *per se* can be proposed as an alternative therapeutic strategy in these patients. Moreover, the response to Tamoxifen induced the best response in the context of one of the ER+ PDXs (MBC3), likely depending on the cell sensitivity due to ESR1 expression.

Finally, we combined the standard and targeting drugs, converging on the aforementioned pathways activation for the onset of resistance mechanisms. In our PDX cells, Paclitaxel and Everolimus combination induced the best response, since Everolimus reversed chemotherapy-resistance and greatly increased the apoptotic effects, confirming the additive effects previously described with other PI3K-mTOR inhibitors, although a significant effect on patients’ survival remains to be elucidated [62,63]. Similar successful combinations of Everolimus and chemotherapy have been described in other metastatic tumors (i.e., large cell neuroendocrine lung carcinoma, gastroesophageal carcinoma, and pancreatic cancer) [64,65,66], thus supporting the relevance of combining targeted and standard drugs to improve therapeutic efficacy. Concerning the Everolimus-based treatment in ER+ samples, our PDXs showed a better response in combination with endocrine therapy. The efficacy of this therapeutic regimen was confirmed by the results that were obtained in the TAMRAD open-label trial, in which the mTOR inhibitor Everolimus combined with tamoxifen reversed endocrine resistance, thus inducing substantially longer patients’ survival [67].

Cell death was also obtained by the convergence of TP53 inhibition and the use of genotoxic agents (i.e., Paclitaxel), either in TP53 -wild type or, surprisingly, -mutant PDXs. In vitro and in vivo studies have shown that the TP53-inhibitors are less toxic in normal cells [68]. Moreover, the ability of TP53-small molecules inhibitor to synergize with conventional chemotherapeutic agents has been well established in other tumor types, as in leukemia, ovarian cancer, and non-small lung cancer [69,70,71], thus suggesting the possibility to reduce chemotherapeutic doses in clinical settings minimizing any potential side-effects in cancer patients [56].

In conclusion, our study supports the idea that the extensive use of PDX models will bring significant advantages to the clinically predictive value of genomic and transcriptomic biomarkers. In this approach, screening for a response to classic cytotoxic and alternative-target agents can be performed and novel combination strategies can be proposed. Moreover, this model is useful for saving time for therapeutic decision and reflects personalized medicine approach in a preclinical setting. Further in vivo studies are necessary to take the impact on therapy regimens of either the microenvironment or the immune system into consideration, since the cells have been grown in immunocompromised hosts, as well as the possibility of side-effects due to drug toxicities.

## Supplementary Materials

The following are available online at https://www.mdpi.com/2073-4409/8/6/605/s1, Figure S1. PDX recapitulates molecular patients’ features.; Figure S2. Breast cancer PDXs recapitulate patients’ genomic alterations.; Figure S3. Activation of oncogenic pathways is revealed by transcriptomic profile in PDXs.; Figure S4. Response of PDX cells to standard drugs administration parallels patients’ sensitivity.; Figure S5. Response of PDX cells to drug administration resemble specific drug effects. Supplementary Table S1. Patients’ tumors transplanted in mice and corresponding positivity of engraftment. Supplementary Table S2. Genomic and transcriptomic profiles reveal high heterogeneity among PDXs. Supplementary Methods.

## References

[1] Ginsburg O., Bray F., Coleman M.P., Vanderpuye V., Eniu A., Kotha S.R., Sarker M., Huong T.T., Allemani C., Dvaladze A. (2017). The global burden of women’s cancers: A grand challenge in global health. Lancet.

[2] Sorlie T., Perou C.M., Tibshirani R., Aas T., Geisler S., Johnsen H., Hastie T., Eisen M.B., van de Rijn M., Jeffrey S.S. (2001). Gene expression patterns of breast carcinomas distinguish tumor subclasses with clinical implications. Proc. Natl. Acad. Sci. USA.

[3] Parker J.S., Mullins M., Cheang M.C., Leung S., Voduc D., Vickery T., Davies S., Fauron C., He X., Hu Z. (2009). Supervised risk predictor of breast cancer based on intrinsic subtypes. J. Clin. Oncol..

[4] Perou C.M., Sorlie T., Eisen M.B., van de Rijn M., Jeffrey S.S., Rees C.A., Pollack J.R., Ross D.T., Johnsen H., Akslen L.A. (2000). Molecular portraits of human breast tumours. Nature.

[5] Waks A.G., Winer E.P. (2019). Breast Cancer Treatment: A Review. JAMA.

[6] Onitilo A.A., Engel J.M., Greenlee R.T., Mukesh B.N. (2009). Breast cancer subtypes based on ER/PR and Her2 expression: Comparison of clinicopathologic features and survival. Clin. Med. Res..

[7] Dai X., Li T., Bai Z., Yang Y., Liu X., Zhan J., Shi B. (2015). Breast cancer intrinsic subtype classification, clinical use and future trends. Am. J. Cancer Res..

[8] Johnston S.R. (2015). Enhancing Endocrine Therapy for Hormone Receptor-Positive Advanced Breast Cancer: Cotargeting Signaling Pathways. J. Natl. Cancer Inst..

[9] Hosford S.R., Miller T.W. (2014). Clinical potential of novel therapeutic targets in breast cancer: CDK4/6, Src, JAK/STAT, PARP, HDAC, and PI3K/AKT/mTOR pathways. Pharmacogenom. Personal. Med..

[10] Palma G., Frasci G., Chirico A., Esposito E., Siani C., Saturnino C., Arra C., Ciliberto G., Giordano A., D’Aiuto M. (2015). Triple negative breast cancer: Looking for the missing link between biology and treatments. Oncotarget.

[11] Sudhan D.R., Schwarz L.J., Guerrero-Zotano A., Formisano L., Nixon M.J., Croessmann S., Ericsson P.I.G., Sanders M., Balko J.M., Avogadri-Connors F. (2019). Extended Adjuvant Therapy with Neratinib Plus Fulvestrant Blocks ER/HER2 Crosstalk and Maintains Complete Responses of ER(+)/HER2(+) Breast Cancers: Implications to the ExteNET Trial. Clin. Cancer Res..

[12] Konieczkowski D.J., Johannessen C.M., Garraway L.A. (2018). A Convergence-Based Framework for Cancer Drug Resistance. Cancer Cell.

[13] Gao H., Korn J.M., Ferretti S., Monahan J.E., Wang Y., Singh M., Zhang C., Schnell C., Yang G., Zhang Y. (2015). High-throughput screening using patient-derived tumor xenografts to predict clinical trial drug response. Nat. Med..

[14] Malaney P., Nicosia S.V., Dave V. (2014). One mouse, one patient paradigm: New avatars of personalized cancer therapy. Cancer Lett..

[15] Dobrolecki L.E., Airhart S.D., Alferez D.G., Aparicio S., Behbod F., Bentires-Alj M., Brisken C., Bult C.J., Cai S., Clarke R.B. (2016). Patient-derived xenograft (PDX) models in basic and translational breast cancer research. Cancer Metastasis Rev..

[16] Bossi D., Cicalese A., Dellino G.I., Luzi L., Riva L., D’Alesio C., Diaferia G.R., Carugo A., Cavallaro E., Piccioni R. (2016). In Vivo Genetic Screens of Patient-Derived Tumors Revealed Unexpected Frailty of the Transformed Phenotype. Cancer Discov..

[17] Kim D., Pertea G., Trapnell C., Pimentel H., Kelley R., Salzberg S.L. (2013). TopHat2: Accurate alignment of transcriptomes in the presence of insertions, deletions and gene fusions. Genome Biol..

[18] Anders S., Pyl P.T., Huber W. (2015). HTSeq—A Python framework to work with high-throughput sequencing data. Bioinformatics.

[19] Anders S., Huber W. (2010). Differential expression analysis for sequence count data. Genome Biol..

[20] Robinson M.D., McCarthy D.J., Smyth G.K. (2010). edgeR: A Bioconductor package for differential expression analysis of digital gene expression data. Bioinformatics.

[21] D’Alesio C., Punzi S., Cicalese A., Fornasari L., Furia L., Riva L., Carugo A., Curigliano G., Criscitiello C., Pruneri G. (2016). RNAi screens identify CHD4 as an essential gene in breast cancer growth. Oncotarget..

[22] Kanaya N., Somlo G., Wu J., Frankel P., Kai M., Liu X., Wu S.V., Nguyen D., Chan N., Hsieh M.Y. (2017). Characterization of patient-derived tumor xenografts (PDXs) as models for estrogen receptor positive (ER+HER2- and ER+HER2+) breast cancers. J. Steroid Biochem. Mol. Biol..

[23] DeRose Y.S., Wang G., Lin Y.C., Bernard P.S., Buys S.S., Ebbert M.T., Factor R., Matsen C., Milash B.A., Nelson E. (2011). Tumor grafts derived from women with breast cancer authentically reflect tumor pathology, growth, metastasis and disease outcomes. Nat. Med..

[24] Li S., Shen D., Shao J., Crowder R., Liu W., Prat A., He X., Liu S., Hoog J., Lu C. (2013). Endocrine-therapy-resistant ESR1 variants revealed by genomic characterization of breast-cancer-derived xenografts. Cell Rep..

[25] Emmanuel N., Lofgren K.A., Peterson E.A., Meier D.R., Jung E.H., Kenny P.A. (2018). Mutant GATA3 Actively Promotes the Growth of Normal and Malignant Mammary Cells. Anticancer Res..

[26] Yates L.R., Knappskog S., Wedge D., Farmery J.H.R., Gonzalez S., Martincorena I., Alexandrov L.B., Van Loo P., Haugland H.K., Lilleng P.K. (2017). Genomic Evolution of Breast Cancer Metastasis and Relapse. Cancer Cell.

[27] Liu J., Li J., Wang H., Wang Y., He Q., Xia X., Hu Z.Y., Ouyang Q. (2019). Clinical and genetic risk factors for Fulvestrant treatment in post-menopause ER-positive advanced breast cancer patients. J. Transl. Med..

[28] Lefebvre C., Bachelot T., Filleron T., Pedrero M., Campone M., Soria J.C., Massard C., Levy C., Arnedos M., Lacroix-Triki M. (2016). Mutational Profile of Metastatic Breast Cancers: A Retrospective Analysis. PLoS Med..

[29] Zhang Y., Kwok-Shing Ng P., Kucherlapati M., Chen F., Liu Y., Tsang Y.H., de Velasco G., Jeong K.J., Akbani R., Hadjipanayis A. (2017). A Pan-Cancer Proteogenomic Atlas of PI3K/AKT/mTOR Pathway Alterations. Cancer Cell.

[30] (2012). Comprehensive molecular portraits of human breast tumours. Nature.

[31] Bruna A., Rueda O.M., Greenwood W., Batra A.S., Callari M., Batra R.N., Pogrebniak K., Sandoval J., Cassidy J.W., Tufegdzic-Vidakovic A. (2016). A Biobank of Breast Cancer Explants with Preserved Intra-tumor Heterogeneity to Screen Anticancer Compounds. Cell.

[32] Han L., Li L., Wang N., Xiong Y., Li Y., Gu Y. (2018). Relationship of Epidermal Growth Factor Receptor Expression with Clinical Symptoms and Metastasis of Invasive Breast Cancer. J. Interferon Cytokine Res..

[33] Wang D.Y., Gendoo D.M.A., Ben-David Y., Woodgett J.R., Zacksenhaus E. (2019). A subgroup of microRNAs defines PTEN-deficient, triple-negative breast cancer patients with poorest prognosis and alterations in RB1, MYC, and Wnt signaling. Breast Cancer Res..

[34] Aftab A., Shahzad S., Hussain H.M.J., Khan R., Irum S., Tabassum S. (2019). CDKN2A/P16INK4A variants association with breast cancer and their in-silico analysis. Breast Cancer.

[35] Zhao N., Cao J., Xu L., Tang Q., Dobrolecki L.E., Lv X., Talukdar M., Lu Y., Wang X., Hu D.Z. (2018). Pharmacological targeting of MYC-regulated IRE1/XBP1 pathway suppresses MYC-driven breast cancer. J. Clin. Investig..

[36] Tuo Y., An N., Zhang M. (2018). Feature genes in metastatic breast cancer identified by MetaDE and SVM classifier methods. Mol. Med. Rep..

[37] Yee K.W., Zeng Z., Konopleva M., Verstovsek S., Ravandi F., Ferrajoli A., Ferrajoli A., Thomas D., Wierda W., Apostolidou E. (2006). Phase I/II study of the mammalian target of rapamycin inhibitor everolimus (RAD001) in patients with relapsed or refractory hematologic malignancies. Clin. Cancer Res..

[38] Gelsomino L., Gu G., Rechoum Y., Beyer A.R., Pejerrey S.M., Tsimelzon A., Wang T., Huffman K., Ludlow A., Ando S. (2016). ESR1 mutations affect anti-proliferative responses to tamoxifen through enhanced cross-talk with IGF signaling. Breast Cancer Res. Treat..

[39] Vassilev L.T., Vu B.T., Graves B., Carvajal D., Podlaski F., Filipovic Z., Kong N., Kammlott U., Lukacs C., Klein C. (2004). In vivo activation of the p53 pathway by small-molecule antagonists of MDM2. Science.

[40] Vilgelm A.E., Cobb P., Malikayil K., Flaherty D., Andrew Johnson C., Raman D., Saleh N., Higgins B., Vara B.A., Johnston J.N. (2017). MDM2 Antagonists Counteract Drug-Induced DNA Damage. EBioMedicine.

[41] Chen X., Paudyal S.C., Chin R.I., You Z. (2013). PCNA promotes processive DNA end resection by Exo1. Nucl. Acids Res..

[42] Ballinger T.J., Meier J.B., Jansen V.M. (2018). Current Landscape of Targeted Therapies for Hormone-Receptor Positive, HER2 Negative Metastatic Breast Cancer. Front. Oncol..

[43] Mikula-Pietrasik J., Witucka A., Pakula M., Uruski P., Begier-Krasinska B., Niklas A., Tykarski A., Ksiazek K. (2018). Comprehensive review on how platinum- and taxane-based chemotherapy of ovarian cancer affects biology of normal cells. Cell. Mol. Life Sci..

[44] Xu C., Li X., Liu P., Li M., Luo F. (2019). Patient-derived xenograft mouse models: A high fidelity tool for individualized medicine. Oncol. Lett..

[45] Golan T., Stossel C., Atias D., Buzhor E., Halperin S., Cohen K., Raitses-Gurevich M., Glick Y., Raskin S., Yehuda D. (2018). Recapitulating the clinical scenario of BRCA-associated pancreatic cancer in pre-clinical models. Int. J. Cancer.

[46] Owonikoko T.K., Zhang G., Kim H.S., Stinson R.M., Bechara R., Zhang C., Chen Z., Saba N.F., Pakkala S., Pillai R. (2016). Patient-derived xenografts faithfully replicated clinical outcome in a phase II co-clinical trial of arsenic trioxide in relapsed small cell lung cancer. J. Transl. Med..

[47] Erriquez J., Olivero M., Mittica G., Scalzo M.S., Vaira M., De Simone M., Ponzone R., Katsaros D., Aglietta M., Calogero R. (2016). Xenopatients show the need for precision medicine approach to chemotherapy in ovarian cancer. Oncotarget.

[48] Francis O.L., Milford T.A., Beldiman C., Payne K.J. (2016). Fine-tuning patient-derived xenograft models for precision medicine approaches in leukemia. J. Investig. Med..

[49] Razavi P., Chang M.T., Xu G., Bandlamudi C., Ross D.S., Vasan N., Cai Y., Bielski C.M., Donoghue M.T.A., Jonsson P. (2018). The Genomic Landscape of Endocrine-Resistant Advanced Breast Cancers. Cancer Cell.

[50] Marangoni E., Vincent-Salomon A., Auger N., Degeorges A., Assayag F., de Cremoux P., de Plater L., Guyader C., De Pinieux G., Judde J.G. (2007). A new model of patient tumor-derived breast cancer xenografts for preclinical assays. Clin. Cancer Res..

[51] Wheler J.J., Parker B.A., Lee J.J., Atkins J.T., Janku F., Tsimberidou A.M., Zinner R., Subbiah V., Fu S., Schwab R. (2014). Unique molecular signatures as a hallmark of patients with metastatic breast cancer: Implications for current treatment paradigms. Oncotarget.

[52] Chin L., Andersen J.N., Futreal P.A. (2011). Cancer genomics: From discovery science to personalized medicine. Nat. Med..

[53] Weigelt B., Glas A.M., Wessels L.F., Witteveen A.T., Peterse J.L., van’t Veer L.J. (2003). Gene expression profiles of primary breast tumors maintained in distant metastases. Proc. Natl. Acad. Sci. USA.

[54] Cerk S., Schwarzenbacher D., Adiprasito J.B., Stotz M., Hutterer G.C., Gerger A., Ling H., Calin G.A., Pichler M. (2016). Current Status of Long Non-Coding RNAs in Human Breast Cancer. Int. J. Mol. Sci..

[55] Su X., Malouf G.G., Chen Y., Zhang J., Yao H., Valero V., Weinstein J.N., Spano J.P., Meric-Bernstam F., Khayat D. (2014). Comprehensive analysis of long non-coding RNAs in human breast cancer clinical subtypes. Oncotarget.

[56] Gonzalez-Angulo A.M., Morales-Vasquez F., Hortobagyi G.N. (2007). Overview of resistance to systemic therapy in patients with breast cancer. Adv. Exp. Med. Biol..

[57] Yin S., Bhattacharya R., Cabral F. (2010). Human mutations that confer paclitaxel resistance. Mol. Cancer Ther..

[58] Xu J.H., Hu S.L., Shen G.D., Shen G. (2016). Tumor suppressor genes and their underlying interactions in paclitaxel resistance in cancer therapy. Cancer Cell Int..

[59] Vogelstein B., Lane D., Levine A.J. (2000). Surfing the p53 network. Nature.

[60] Yeung Y., Lau D.K., Chionh F., Tran H., Tse J.W.T., Weickhardt A.J., Nikfarjam M., Scott A.M., Tebbutt N.C., Mariadason J.M. (2017). K-Ras mutation and amplification status is predictive of resistance and high basal pAKT is predictive of sensitivity to everolimus in biliary tract cancer cell lines. Mol. Oncol..

[61] Park J.H., Ryu M.H., Park Y.S., Park S.R., Na Y.S., Rhoo B.Y., Kang Y.K. (2015). Successful control of heavily pretreated metastatic gastric cancer with the mTOR inhibitor everolimus (RAD001) in a patient with PIK3CA mutation and pS6 overexpression. BMC Cancer.

[62] Brana I., Pham N.A., Kim L., Sakashita S., Li M., Ng C., Wang Y., Loparco P., Sierra R., Wang L. (2017). Novel combinations of PI3K-mTOR inhibitors with dacomitinib or chemotherapy in PTEN-deficient patient-derived tumor xenografts. Oncotarget.

[63] Li B., Gu W., Zhu X. (2019). NEAT1 mediates paclitaxel-resistance of non-small cell of lung cancer through activation of Akt/mTOR signaling pathway. J. Drug Target..

[64] Christopoulos P., Engel-Riedel W., Grohe C., Kropf-Sanchen C., von Pawel J., Gutz S., Kollmeier J., Eberhardt W., Ukena D., Baum V. (2017). Everolimus with paclitaxel and carboplatin as first-line treatment for metastatic large-cell neuroendocrine lung carcinoma: A multicenter phase II trial. Ann. Oncol..

[65] Chung V., Frankel P., Lim D., Yeon C., Leong L., Chao J., Ruel N., Luevanos E., Koehler S., Chung S. (2016). Phase Ib Trial of mFOLFOX6 and Everolimus (NSC-733504) in Patients with Metastatic Gastroesophageal Adenocarcinoma. Oncology.

[66] Kordes S., Klumpen H.J., Weterman M.J., Schellens J.H., Richel D.J., Wilmink J.W. (2015). Phase II study of capecitabine and the oral mTOR inhibitor everolimus in patients with advanced pancreatic cancer. Cancer Chemother. Pharmacol..

[67] Baselga J., Campone M., Piccart M., Burris H.A., Rugo H.S., Sahmoud T., Noguchi S., Gnant M., Pritchard K.I., Lebrun F. (2012). Everolimus in postmenopausal hormone-receptor-positive advanced breast cancer. N. Engl. J. Med..

[68] Carvajal D., Tovar C., Yang H., Vu B.T., Heimbrook D.C., Vassilev L.T. (2005). Activation of p53 by MDM2 antagonists can protect proliferating cells from mitotic inhibitors. Cancer Res..

[69] Drakos E., Atsaves V., Schlette E., Li J., Papanastasi I., Rassidakis G.Z., Medeiros L.J. (2009). The therapeutic potential of p53 reactivation by nutlin-3a in ALK+ anaplastic large cell lymphoma with wild-type or mutated p53. Leukemia.

[70] Zanjirband M., Edmondson R.J., Lunec J. (2016). Pre-clinical efficacy and synergistic potential of the MDM2-p53 antagonists, Nutlin-3 and RG7388, as single agents and in combined treatment with cisplatin in ovarian cancer. Oncotarget.

[71] Deben C., Wouters A., Op de Beeck K., van Den Bossche J., Jacobs J., Zwaenepoel K., Peeters M., Van Meerbeeck J., Lardon F., Rolfo C. (2015). The MDM2-inhibitor Nutlin-3 synergizes with cisplatin to induce p53 dependent tumor cell apoptosis in non-small cell lung cancer. Oncotarget.

