# Supplementary material for: Development of Personalized Therapeutic Strategies by Targeting Actionable Vulnerabilities in Metastatic and Chemotherapy-Resistant Breast Cancer PDXs
Source: Cells. 2019 Jun 18;8(6):605. doi: 10.3390/cells8060605 (PMC6627522; doi:10.3390/cells8060605)
Supplement: Supplementary file 1 [file cells-08-00605-s001.zip › cells-502088-SI/Cells_Supplementary information.pdf]

Supplementary information

Cell lines. MCF10DCIS.com were purchased from Wayne State University, 5057 Woodward Avenue, Detroit – Michigan; MCF7 from NIH. Cell lines were maintained in their respective media as recommended by suppliers. Cell line authentication was performed in house by Gene Print 10 System every six months (Promega). All cell lines were tested for mycoplasma and resulted negative.

Immunohistochemistry. Tumor fragments from patients and corresponding PDXs were formalin-fixed and paraffin-embedded. After deparaffinization, sections were treated with citrate for 50 minutes at 95°C, followed by incubation with 3% hydrogen peroxide in distilled water for 5 minutes at RT. Sections were stained with Estrogen, Progesterone, HER2 and Ki67 (all from Dako) primary antibodies. Images were acquired by Olympus BX51 up-right (objective 40x) connected to Nikon Color Camera (software NIS-elements). Tumors were characterized on the basis of the prognostic clinical markers by pathologists and compared to patient tumors. Other antibodies used for characterization were HLA [EMR8-5] (Abcam), α-SMA (Abcam) and Pan-cytokeratin (Abcam), TP53 (Santa Cruz Biotechnology), PI3KCA (Upstate).

Mutation frequency analysis on MBCproject Data. Analysis on Metastatic Breast Cancer (MBC) project data set of breast cancer patients was performed by using publicly available data in cBioportal for Cancer Genomics by considering mutation percentage of genes of interest from whole-exome sequencing of 237 patients with metastatic breast cancer. Moreover, annotation from OncoKB were considered for the prediction on clinical implication and biological effects.
