# Supplementary material for: Development of Personalized Therapeutic Strategies by Targeting Actionable Vulnerabilities in Metastatic and Chemotherapy-Resistant Breast Cancer PDXs
Source: Cells. 2019 Jun 18;8(6):605. doi: 10.3390/cells8060605 (PMC6627522; doi:10.3390/cells8060605)
Supplement: Supplementary file 1 [file cells-08-00605-s001.zip › cells-502088-SI/Supplfigures.pdf]

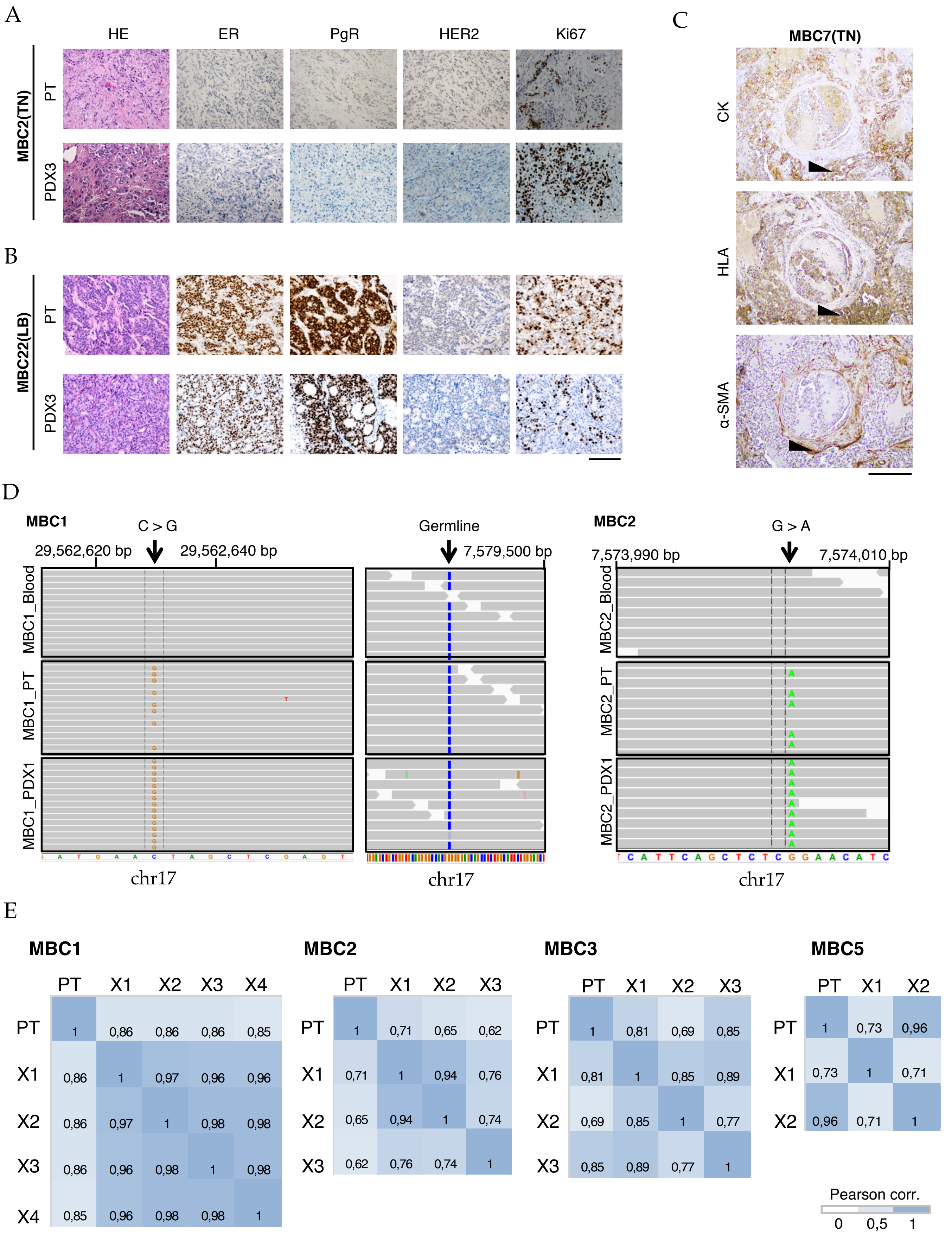

MBC1	PT	X1	X2	X3	X4
PT	1	0,86	0,86	0,86	0,85
X1	0,86	1	0,97	0,96	0,96
X2	0,86	0,97	1	0,98	0,98
X3	0,86	0,96	0,98	1	0,98
X4	0,85	0,96	0,98	0,98	1

MBC2	PT	X1	X2	X3
PT	1	0,71	0,65	0,62
X1	0,71	1	0,94	0,76
X2	0,65	0,94	1	0,74
X3	0,62	0,76	0,74	1

MBC3	PT	X1	X2	X3
PT	1	0,81	0,69	0,85
X1	0,81	1	0,85	0,89
X2	0,69	0,85	1	0,77
X3	0,85	0,89	0,77	1

MBC5	PT	X1	X2
PT	1	0,73	0,96
X1	0,73	1	0,71
X2	0,96	0,71	1

Supplementary Figure S1

Figure S1. PDX recapitulates molecular patients' features. (A-B) Representative histology by hematoxylin-eosin and immunoistochemistry (IHC) for estrogen (ER), progesterone (PgR), HER2 and Ki67 for one TN **(A)** and one LB **(B)** patient (PT) and the corresponding PDX at their third passage. Scale bar: 250 μm. **(C)** IHC for cytokeratin, Human Leukocyte Antigen (HLA) and Alpha (α-) Smooth Muscle Antibody (SMA) in one of the PDX. Scale bar: 50 μm. **(D)** Representative snapshots visualized on IGV of chromosome (chr) 17 obtained from exome sequencing of blood (normal counterpart), tumors and PDXs derived from two PTs (MBC1 and MBC2). The sites of the somatic or germline mutations are indicated by the arrows. (**E**) Heatmaps represent Pearson correlation (r) of Variant Allele Frequency for PT and corresponding PDX at diverse passages in mice.

A

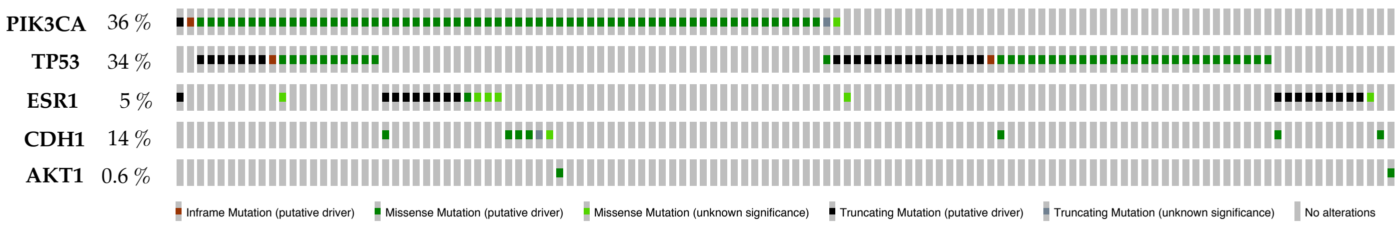

B

Gene	Sample	Chr	Start_position	End_position	Ref	Alt	Mutation	Protein change	Clinical implication	Biological effect
**TP53**	MBC7	chr17	7578271	7578271	T	C	Missense_Mutation	p.H193R	Likely oncogenic	Likely loss of function
	MBC18	chr17	7577574	7577574	T	C	Missense_Mutation	p.Y236C		
	MBC21	chr17	7577088	7577088	T	A	Missense_Mutation	p.T284S		
	MBC21	chr17	7577086	7577087	-	GGGAGA	Insertion	p.284_285insLP		
	MBC11	chr17	7577094	7577094	G	A	Missense_Mutation	p.R282W		
	MBC5	chr17	7577105	7577105	G	A	Missense_Mutation	p.P278L	Likely oncogenic	Loss of function
	MBC2	chr17	7574003	7574003	G	A	Nonsense_Mutation	p.R342*	Likely oncogenic	Likely loss of function
**PIK3CA**	MBC7	chr3	178952074	178952074	G	A	Missense_Mutation	p.M1043I	Oncogenic	Gain of function
	MBC7	chr3	178921549	178921549	T	A	Missense_Mutation	p.V344E		
	MBC22	chr3	178948136	178948136	G	A	Missense_Mutation	p.E970K	Predicted oncogenic	unknown
	MBC22	chr3	178952085	178952085	A	G	Missense_Mutation	p.H1047R	Oncogenic	Gain of function
	MBC11	chr3	178936082	178936082	G	A	Missense_Mutation	p.E542K	Oncogenic	Gain of function
	MBC5	chr3	178952084	178952084	C	T	Missense_Mutation	p.H1047Y	Oncogenic	Gain of function
**ESR1**	MBC22	chr6	152419923	152419923	A	C	Missense_Mutation	p.Y537S	Oncogenic	Gain of function
**AKT1**	MBC7	chr14	105239217	105239217	C	G	Missense_Mutation	p.Q85H		
**CDH1**	MBC7	chr16	68842395	68842395	G	C	Missense_Mutation	p.Q152H		

C

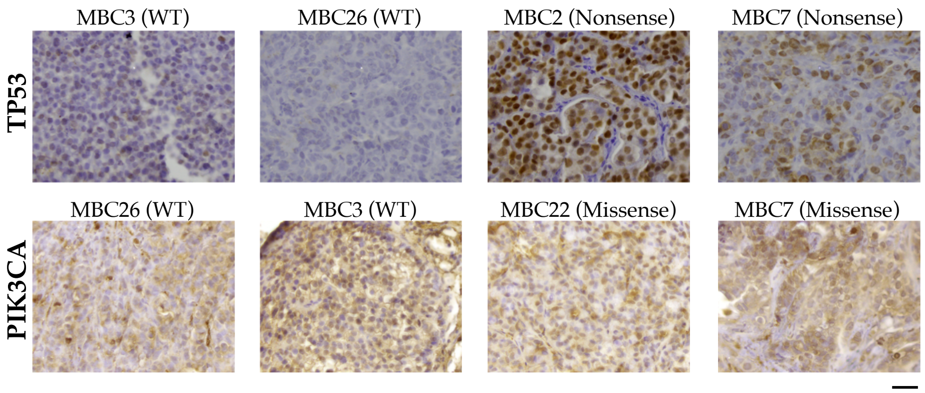

Supplementary Figure S2

Figure S2. Breast cancer PDXs recapitulate patients' genomic alterations. (A) Genetic alterations of PIK3CA, TP53, ESR1, CDH1 and AKT1 in metastatic breast cancer patients registered in Metastatic Breast Cancer Project database (http://www.cbioportal.org). Percentage and kind of mutations are reported. **(B)** List of genetic alterations for aforementioned genes in each PDX. Gene, chromosome (chr), start and end position, annotated mutation and protein changes are reported. Clinical implications and biological effects (if available) of each alteration with respect to specific protein changes are reported as in cbioportal.org. **(C)** IHC evaluation of TP53 and PIK3CA protein expression in some of the PDXs is reported. Counterstain was performed by haematoxylin. Scale bar: 50 μm.

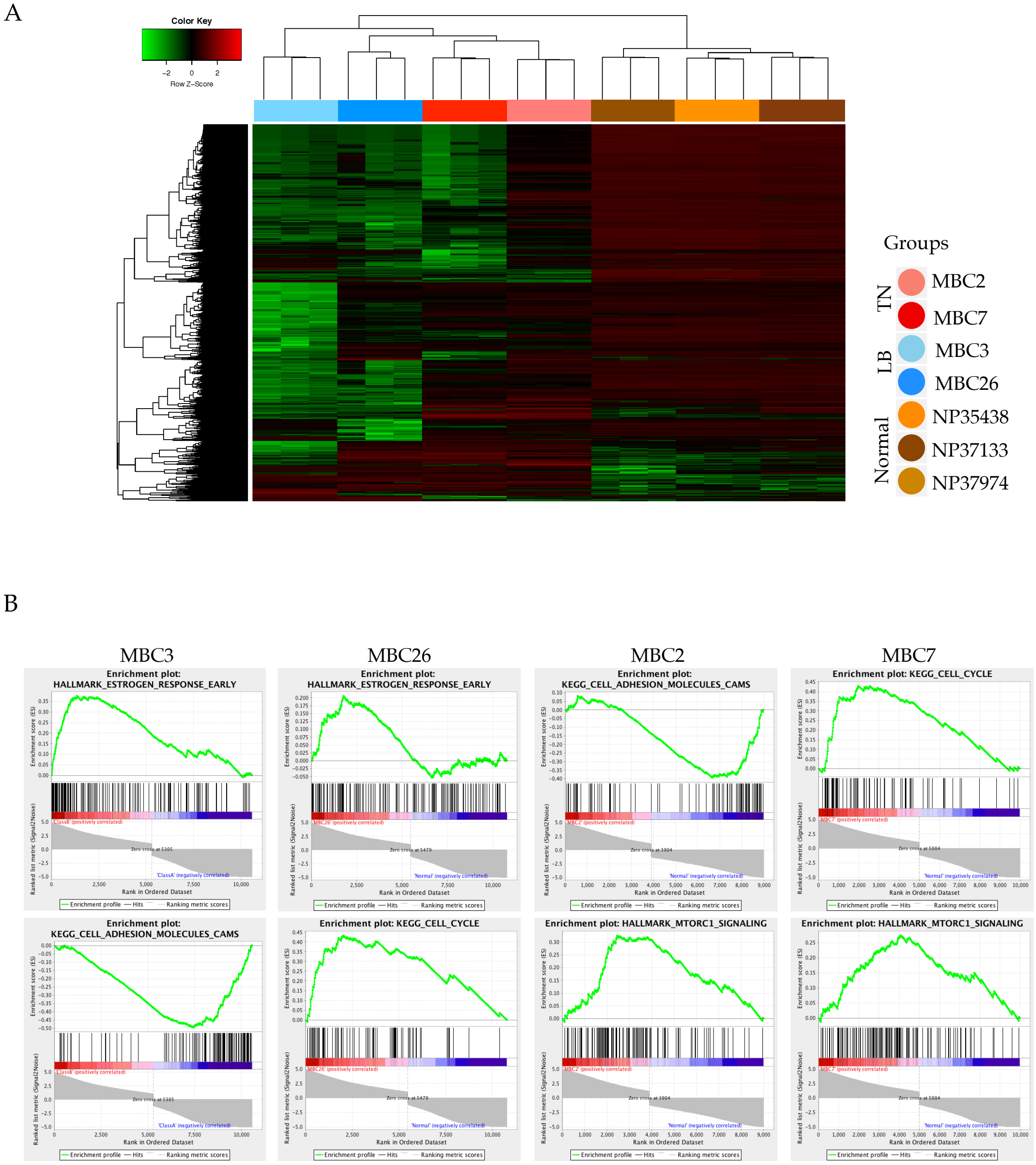

Figure S3. Activation of oncogenic pathways is revealed by transcriptomic profile in PDXs. (A) Hierarchical clustering of 1869 most variable genes in 3 Normal breast, 2 LB and 2 TN breast cancer PDXs (n=3 replicates). **(B)** Enrichment plots for some representative functions in PDXs by Gene Set Enrichment Analysis (GSEA) are reported.

A

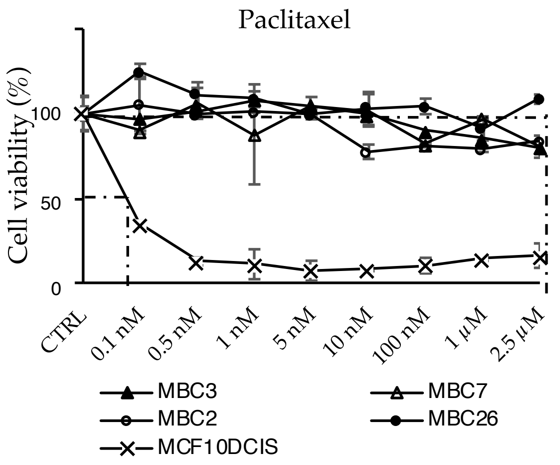

B

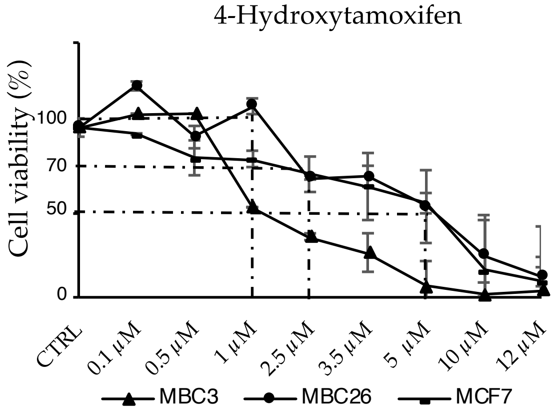

Figure S4. Response of PDX cells to standard drugs administration parallels patients' sensitivity. Effects on *in vitro* cells viability (expressed as %) due to standard therapies is reported for each PDX. **(A)** Paclitaxel (PTX) was tested in 2 TN (MBC2 and MBC7) and 2 LB (MBC3 and MBC26) PDXs (n=3; mean ± SD). **(B)** 4-Hydroxytamoxifen (4-OHT) treatment at increasing concentrations was tested in 2 LB (MBC3 and MBC26) PDX cells (n=3; mean ± SD). MCF10DCIS cell line was used as positive control for PTX treatment; MCF-7 as positive control for 4-OHT treatment. Dotted lines represent IC30 (70% viability), IC50 (50% viability) or no effect (100% viability).

A

	STANDARD		ALTERNATIVE		COMBINATORIAL (PTX)		COMBINATORIAL (TAM)	
	PTX	4-OHT	IDAS	EVER	PTX+IDAS	PTX+EVER	4-OHT+IDAS	4-OHT+EVER
MBC2	R	-	R	IC50: 10 μM	P<0.05	P<0.001	-	-
MBC7	R	-	R	IC30: 10 μM	P<0.001	P<0.001	-	-
MBC3	R	IC50: 1 μM	IC30: 15 μM	IC50: 20 μM	P<0.001	P<0.001	NS	P=0.0578
MBC26	R	IC50: 5 μM	IC30: 15 μM	IC50: 15 μM	P<0.001	P<0.001	P<0.05	P<0.05

B

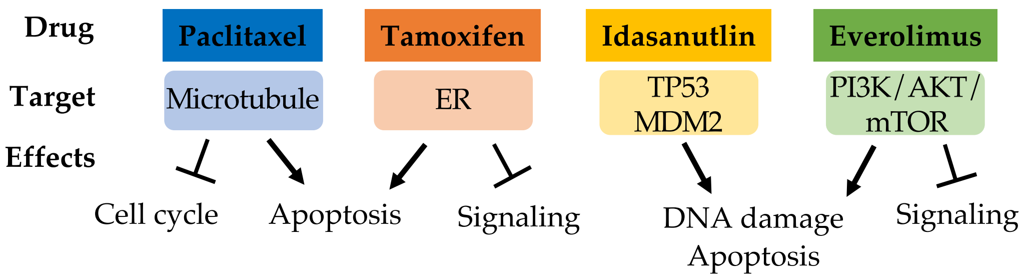

C

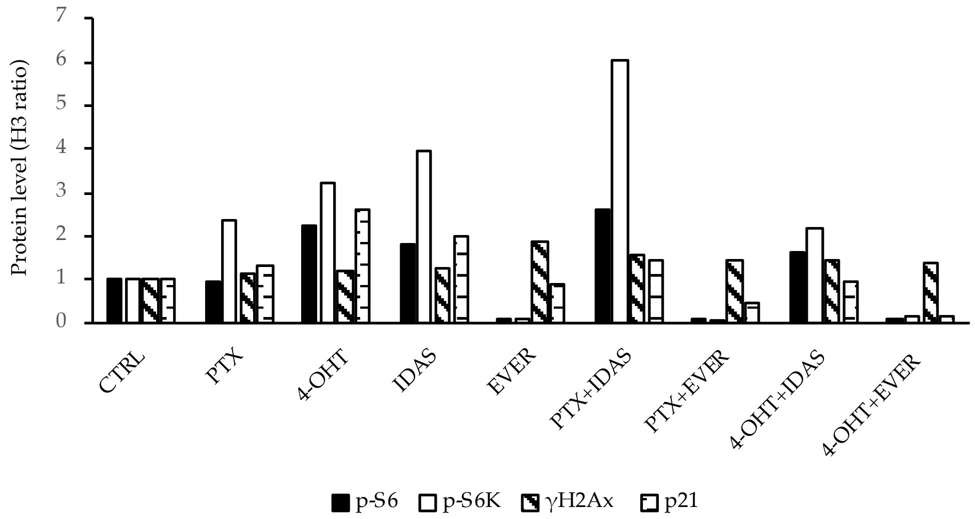

Figure S5. Response of PDX cells to drug administration resembles specific drug effects. (A) Table summarizes resistance (R) or response with respect to IC30 or IC50 values for standard (Paclitaxel – PTX- or 4-Hydroxytamoxifen – 4-OHT) or alternative (Idasanutlin – IDAS - or Everolimus - EVER) therapies. P values of significative differences of cell viability among standard and combinatorial (standard + alternative) drugs administration were calculated by one-way ANOVA followed by Dunnett Post test. NS: not significant. **(B)** Scheme represents targets and biological effects reported for each drug administration. **(C)** Protein levels of mTOR1 target – phospho-S6(p-S6) and phospho-S6K (p-S6K), γH2Ax and p21 in response to aforementioned treatments, were quantified with respect to H3 as normalizer, relatively to the control (CTRL - no treatment).
