# Supplementary material for: Development of Personalized Therapeutic Strategies by Targeting Actionable Vulnerabilities in Metastatic and Chemotherapy-Resistant Breast Cancer PDXs
Source: Cells. 2019 Jun 18;8(6):605. doi: 10.3390/cells8060605 (PMC6627522; doi:10.3390/cells8060605)
Supplement: Supplementary file 1 [file cells-08-00605-s001.zip › cells-502088-SI/Table S1.pdf]

Table S1. Patients' tumors transplanted in mice and corresponding positivity of engraftment. For each patient with metastatic breast cancer (MBC) site of metastasis, year of surgery and molecular subtype (LB: Luminal B; TN: Triple Negative; HER2+: HER2 positive) is reported. For each Patient Derived Xenografts (PDXs) positivity (YES) or negativity (NO) to the engraftment and molecular subtype is reported.

	Patient			PDXs	
	**Metastasis**	**Diagnosis**	**Subtype**	**Engraftment**	**Subtype**
**MBC4**	liver	2012	LB	YES	LB
**MBC7**	liver	2012	TN	YES	TN
**MBC12**	liver	2012	LB	NO	-
**MBC14**	liver	2012	HER2+	NO	-
**MBC18**	liver	2012	LB	YES	LB
**MBC19**	liver	2012	LB	NO	-
**MBC20**	liver	2012	LB	NO	-
**MBC21**	liver	2012	HER2+	YES	HER2+
**MBC22**	liver	2012	LB	YES	LB
**MBC24**	liver	2012	LB	NO	-
**MBC25**	liver	2012	LB	NO	-
**MBC26**	liver	2012	LB	YES	LB
**MBC28**	node	2012	TN	NO	-
**MBC1**	lung	2013	TN	YES	TN
**MBC2**	lung	2013	TN	YES	TN
**MBC3**	lung	2013	LB	YES	LB
**MBC16**	lung	2013	LB	NO	-
**MBC5**	lung	2013	LB	YES	LB
**MBC6**	lung	2013	HER2+	NO	-
**MBC8**	node	2013	LB	NO	-
**MBC9**	node	2013	TN	NO	-
**MBC10**	lung	2014	LB	YES	LB
**MBC11**	lung	2014	LB	YES	LB

Table S2. Genomic and transcriptomic profiles reveal high heterogeneity among PDXs (see excel file)**.** Exome sequencing analysis from 4 metastatic breast cancer Patient (PT) and corresponding Patient-derived xenograft (PDX) at different passages reveals high resemblance of mutational profile. Reference was obtained by normal blood samples. Chromosome (chr), start and end position, reference and alteration, variant allele frequency (>0.05 at least in one sample), gene name and variants classification are reported. For Insertions or Deletions, presence (YES) or absence (NO) of alterations is indicated for each sample.
Exome sequencing analysis from 12 metastatic breast cancer PDXs reveals mutation profiles of breast cancer tumors (see excel file). Gene name, chromosome (chr), start and end position, reference and alteration, type of annotated mutation and protein change are reported for each PDX.
RNA-seq analysis of PDXs highlighted a different expression pattern in each PDX (see excel file). Differential expression analysis for 4 metastatic breast cancer PDXs with respect to the normal breast tissue (n=3 samples) is reported. FDR≤0.001 and log2 fold change (FC) ≥ |2|.
